# Responsiveness and minimal clinically important difference of EQ-5D-5L in patients with coronary heart disease after percutaneous coronary intervention: A longitudinal study

**DOI:** 10.3389/fcvm.2023.1074969

**Published:** 2023-03-09

**Authors:** Yu Zheng, Lei Dou, Qiang Fu, Shunping Li

**Affiliations:** ^1^Center for Health Management and Policy Research, School of Public Health, Cheeloo College of Medicine, Shandong University, Jinan, Shandong, China; ^2^NHC Key Lab of Health Economics and Policy Research (Shandong University), Jinan, Shandong, China; ^3^Center for Health Preference Research, Shandong University, Jinan, Shandong, China; ^4^Department of Cardiovascular Surgery, General Hospital of Tianjin Medical University, Tianjin, China

**Keywords:** responsiveness, minimal clinically important difference, minimal detectable change, EQ-5D-5L, coronary heart disease, percutaneous coronary intervention

## Abstract

**Background:**

Although the five-level version of the EuroQol five-dimensional questionnaire (EQ-5D-5L) has been validated in various diseases, no empirical study has evaluated the responsiveness and minimal clinically important difference (MCID) of the instrument in patients with coronary heart disease (CHD), which limits the interpretability and clinical application of EQ-5D-5L. Therefore, this study aimed to determine the responsiveness and MCID of EQ-5D-5L in patients with CHD who underwent percutaneous coronary intervention (PCI) and identify the relationship between the MCID values and minimal detectable change (MDC).

**Methods:**

Patients with CHD were recruited for this longitudinal study at the Tianjin Medical University’s General Hospital in China. At baseline and 4 weeks after PCI, participants completed the EQ-5D-5L and Seattle Angina Questionnaire (SAQ). Additionally, we used the effect size (ES) to assess the responsiveness of EQ-5D-5L. The anchor-based, distribution-based, and instrument-based methods were used in this study to calculate the MCID estimates. The MCID estimates to MDC ratios were computed at the individual and group levels at a 95% CI.

**Results:**

Seventy-five patients with CHD completed the survey at both baseline and follow-up. The EQ-5D-5L health state utility (HSU) improved by 0.125 at follow-up compared with baseline. The ES of EQ-5D HSU was 0.850 in all patients and 1.152 in those who improved, indicating large responsiveness. The average (range) MCID value of the EQ-5D-5L HSU was 0.071 (0.052–0.098). These values can only be used to determine whether the change in scores were clinically meaningful at the group level.

**Conclusion:**

EQ-5D-5L has large responsiveness among CHD patients after undergoing PCI surgery. Future studies should focus on calculating the responsiveness and MCID for deterioration and examining the health changes at the individual level in CHD patients.

## 1. Introduction

Coronary heart disease (CHD) is one of the most common cardiovascular diseases worldwide. Despite tremendous efforts in the prevention, treatment, and rehabilitation of CHD, which has led to a decline in mortality over the last few decades, CHD remains the leading cause of death and disability in adults worldwide, including in China (1, 2). It is expected to account for 30.5% of all deaths worldwide by 2030 (2). In China, it is estimated that approximately 11 million people have CHD, and the morbidity and mortality of CHD are still increasing annually (3).

Several factors are thought to increase the likelihood of CHD development. The main traditional risk factors are hypertension, smoking, hyperlipidemia, diabetes, old age, gender, obesity, lack of exercise, and family history of CHD (4). Often, these risk factors do not exist in isolation. Therefore, CHD patients commonly present with multiple comorbidities and are often accompanied by psychological problems such as anxiety and depression (5). Percutaneous coronary intervention (PCI) guided by coronary physiology can relieve patients’ clinical symptoms and has been regarded as a standard and effective treatment for CHD (6, 7). Previous studies have found that PCI can improve the health-related quality of life (HRQoL) of patients with CHD, but the effects do not last (8).

HRQoL refers to different health domains, including physiological, psychological, and social components (9). It is increasingly accepted as an important outcome, especially for chronic conditions, including CHD (8, 10). HRQoL in patients with CHD can be quantified using generic or disease-specific instruments (11). CHD-specific HRQoL instruments, such as the Seattle Angina Questionnaire (SAQ) and the Minnesota Living with Heart Failure Questionnaire (MLHF), can examine the specific impact of CHD (11). Generic instruments, such as SF-36 and EQ-5D, enable the comparison of HRQoL between CHD and other conditions (11).

The EuroQoL five-dimensional questionnaire (EQ-5D) is a generic preference-based HRQoL instrument developed by the EuroQoL group that has been widely used and validated in populations with various diseases (12). The original version of EQ-5D has three response levels (13). However, studies have found that the three-level version has an obvious ceiling effect and cannot capture small changes sufficiently (14, 15). Therefore, a five-level version of EQ-5D was developed (16). Furthermore, health state utility (HSU) generated by the EQ-5D can support the calculation of quality-adjusted life years (QALYs), which will allow for pharmacoeconomic evaluation (17).

However, the interpretation of HRQoL instrument scores faces enormous challenges, particularly in defining what constitutes a trivial or an important change in patients’ quality of life (18–20). The results of HRQoL scores are usually analyzed and interpreted using statistical tests in clinical research. Although statistical tests can reflect statistical changes in the measured outcomes, they do not always indicate that the changes are clinically relevant (18, 21). Therefore, to explain the clinical relevance of score changes, the concept of minimal clinically important difference (MCID) was developed (19). Calculating the MCID values of HRQoL instruments can not only assess clinically meaningful score changes for patients in clinical trials but also supports the interpretability of the measuring instrument (22).

The minimal detectable change (MDC) is the smallest change in scores that can be detected after considering the measurement error (21). For a reliable instrument, the MCID should be greater than MDC (23, 24). Therefore, we can use the ratio between the MCID and MDC to judge whether the calculated MCID is a real change or just a meaningless measurement error, which can support the application of MCID in clinical research. Furthermore, MCID and MDC are related to responsiveness. The former two indicators are more clinically oriented, and the responsiveness is regarded as an indicator reflecting the ability of the instrument to detect changes over time (25, 26).

Previous studies have shown that the responsiveness and MCID are affected by clinical setting, and their use requires calculations in specific disease contexts (27, 28). Therefore, although the responsiveness and MCID of the EQ-5D-5L HSU have been calculated in patients with various diseases, the results vary. For example, previous studies have found small-to-moderate responsiveness in patients with stroke (29) and large responsiveness in venous leg ulcers (30). The MCID of the EQ-5D-5L HSU in patients with cervical intraepithelial neoplasia (CIN) was 0.039 (31), while in patients with COPD was 0.051 (32). Furthermore, a study evaluating the relationship between MCID and MDC in patients with CIN showed that when the MCID of the EQ-5D HSU was 0.039, it could not be distinguished from measurement error at the individual level (31). Another study in patients after hip or knee replacement found that when the MCID of EQ-5D HSU was 0.32, it could be distinguished from measurement error at both individual and group levels (33). All of these studies support the interpretability and applicability of EQ-5D in different clinical settings. Nevertheless, according to our knowledge, no empirical study has evaluated the responsiveness and MCID of EQ-5D-5L in CHD patients after PCI, and none has analyzed the relationship between MCID and MDC in CHD patients.

Therefore, our study aimed to (1) evaluate the responsiveness of EQ-5D-5L in CHD patients who underwent PCI, (2) calculate the MCID estimates of EQ-5D-5L HSU, and (3) identify the validity of MCID by using the ratios between MCID and MDC.

## 2. Materials and methods

### 2.1. Study design and population

This prospective cohort study was conducted at the General Hospital of Tianjin Medical University, China, between April and September 2019. Inclusion criteria were (1) recruited from the cardiology inpatients, (2) one or more lesions with ≥50% stenosis as shown by coronary angiography and met the requirements of the “Chinese Guidelines for Percutaneous Coronary Intervention” (34), (3) aged 18 years or older, and (4) will undergo PCI within 1 or 2 days. The exclusion criteria were (1) unwillingness to provide informed consent, (2) inability to understand the questionnaire, (3) serious comorbidities (such as severe liver and kidney insufficiency or cirrhosis, malignant tumor), (4) a history of mental illness, and (5) hearing or vision impairment.

The sample size was considered sufficient if the number of patients was at least 5–10 times the number of items in the main outcome (EQ-5D-5L in this study). Depending on the questionnaire used in the study, the estimated sample size was at least 25–50 cases. We anticipated a potential 20% loss to follow-up, so at least 60 patients were required. All patients who completed the questionnaire and provided informed consent were enrolled in this study. Ethical approval was obtained from the Ethics Review Board of the School of Health Care Management, Shandong University (No. ECSHCMSDU20191002). The study was carried out in accordance with the Declaration of Helsinki.

### 2.2. Study procedure and quality control

Before the survey, we provided homogeneous training to all investigators, addressing the survey process and questionnaires, the significance of questionnaire items, and points for attention during the survey. During the survey process, uniform terminology was adopted to explain the research purpose and questionnaire requirements to all participants in detail. Participants filled out the questionnaire anonymously after they provided their informed consent.

All eligible participants completed the questionnaires at baseline (before PCI surgery) and a follow-up point (4-week after PCI). At baseline, socio-demographic characteristics, including age, gender, marital status, education level, and occupation, were collected through face-to-face interviews with eligible patients on-site. Additionally, clinical data, including CHD types, duration time, disease status, and comorbidities, were collected from their clinicians. Well-trained investigators conducted a follow-up survey over the telephone. The participants’ phone numbers were randomly and anonymously assigned to different investigators. After confirming the free time of each participant, the investigator conducted a one-to-one telephone follow-up. At both time points, the participants were asked to complete the SAQ and EQ-5D-5L to evaluate their health status. The questionnaire was completed within 10–20 min. The data were entered uniformly. After the original data were entered, another member checked them to ensure the correctness of the data entry.

### 2.3. Instruments

EQ-5D-5L includes a multi-attribute health description system based on five dimensions: mobility, self-care, usual activities, pain/discomfort, and anxiety/depression (16). Each dimension has a five-point response option: no problems, slight problems, moderate problems, severe problems, and extreme problems (35). The Chinese version of the EQ-5D-5L has been verified, and the Chinese-specific tariff was used in this study (35, 36). EQ-5D-5L HSU calculated by Chinese-specific tariff ranges from −0.391 to 1.000, where 1 indicates “full health” and 0 means “dead.” A negative HSU represents a certain health status that is worse than death.

Seattle Angina Questionnaire is a disease-specific instrument used to measure the health status of CHD patients. It consists of 19 items divided into five domains: physical limitation, angina stability, angina frequency, treatment satisfaction, and disease perception (37). The SAQ was scored by assigning each response an ordinal value, with 1 being the option with the lowest level of health and 5 being the option with the best level of health. All domain scores can be calculated by summing the scores of items in each domain and can be transformed to 0–100 points. Higher scores indicated a better quality of life (38). The Chinese version of the SAQ has been proven to be a valid and reliable instrument (39).

### 2.4. Statistical analysis

Descriptive statistics were used to describe the socio-demographic characteristics, clinical characteristics, and distribution of EQ-5D-5L HSU and SAQ scores. Characteristics were presented as means and standard deviations (SD) for continuous variables and numbers and percentages (%) for categorical variables. For comparing scores between two-time points, the paired t-test was used for parametric data, and the Wilcoxon signed-rank test was used for nonparametric data. Statistical analysis was conducted using the SPSS software (IBM SPSS Statistics 25.0). All statistical tests were two-tailed, with a significance level of 0.05.

### 2.5. Responsiveness

Researchers have no consensus regarding the best method for calculating responsiveness (40, 41). Therefore, we used effect size (ES) to examine the responsiveness in this study. ES was defined as the difference in scores between baseline and follow-up divided by the SD of the baseline scores (21, 29, 42). According to Cohen’s d criteria, ES can be categorized as small (<0.5), moderate (0.5 ~ 0.8), or large (>0.8) (43).

### 2.6. Minimal clinically important difference

**Anchor-based method**: The anchor-based approach uses external criteria to anchor minimal but important change scores for participants (44). Correlation ≥|0.3| can be used as a threshold to assess the usefulness of the anchor (44). Therefore, Spearman’s correlation coefficient was used to quantify the association between changes in EQ-5D-5L HSU and SAQ scores. We used half the SD of anchor scores at baseline as the lower cut-off of minimal change and twice the lower cut-off as the upper cut-off (45, 46). Specifically, participants were categorized into three groups according to the change scores of the anchor: no change (<0.5 baseline SD), minimal change (≥0.5 baseline SD and ≤1 baseline SD), and large change (>1 baseline SD). To obtain the MCID for improvement, the mean change score for the “minimal improvement” group was subtracted from the average change score for the “no change” group (47). Likewise, the deteriorative MCID was the difference between the mean change score for the “minimal deterioration” group and the “no change” group (47).

A receiver operating characteristic (ROC) curve was constructed to estimate the MCID in this study, and the area under the curve (AUC) was used to represent the ability of the instrument to distinguish patients who underwent a clinically meaningful change. The Youden index was calculated to determine MCID estimates with the highest sensitivity and specificity using the following formula: $\mathrm{Youden index}=\mathrm{sensitivity}-\left(1-\mathrm{specificity}\right).$ The cut-off point corresponding to the maximum Youden index was the optimal cut-off value of the ROC curve and was the MCID (48, 49).

**Instrument-based method**: Instrument-based method is based on the average difference in the EQ-5D HSU between the baseline health states and single-level transitions to other health states (50). According to the direction of single-level transitions, MCID can be categorized into three groups: only transitions to better health states, only transitions to worse states and all transitions to other health states (46). In this study, we only focused on the better transitions. In addition, because the conversion parameter between Lever 3 (moderate problem) and Level 4 (severe problem) exceeds other adjacent levers at least 1.4 times in all dimensions according to the Chinese scoring algorithm, we excluded the interconversion between these two levels to avoid overestimating MCID (31, 46). Finally, when calculating the improved MCID, we excluded the baseline health state “11,111” because it could no longer be improved.

**Distribution-based method**: Based on previous studies, half the SD of baseline scores and one-SEM were considered to approximate values of MCID (45, 51, 52). Therefore, we calculated half the SD of the EQ-5D-5L HSU at baseline as the MCID. Additionally, the standard error measurement (SEM) was calculated using the following formula: $\mathrm{SEM}=\mathrm{SD}\sqrt{\left(1-r\right)}$ , where r is the test–retest reliability or Cronbach’s α coefficient (51). The Cronbach’s α coefficient of the EQ-5D-5L was calculated at baseline and follow-up in this study. The SEM was computed for the baseline and follow-up scores, and the mean was calculated to provide an MCID estimate.

### 2.7. Minimal detectable change

The MDC is derived from SEM and is calculated using the formula: $\mathrm{MDC}=\mathrm{SEM}*Z-\mathrm{score}*\sqrt{2}\;$ (33). In this study, a 95% confidence level was established, corresponding to a *Z*-score of 1.96. This MDC was considered MDC_95%(ind)_, representing the smallest detectable change after considering the measurement error at the individual level. According to Boer, MDC_95%(group)_ is equal to MDC_95%(ind)_ divided by $\sqrt{n}$ , where n represents the sample size (53). Finally, we used the ratio of MCID to MDC for comparisons at the individual and group levels. If the ratio of MCID to MDC > 1, the calculated MCID can be used to reflect the real minimal important change. Otherwise, the calculated MCID represents the measurement error of the questionnaire and is not a valid value (54).

## 3. Results

### 3.1. Descriptive analysis

Seventy-nine patients were included at baseline. Three patients were lost to follow-up during the study period, and one died. The socio-demographic and clinical characteristics of the 75 patients who completed the questionnaire at both time points are presented in Table 1. The mean age of the patients was 64.6 ± 9.1 years. Most of them were male (62.7%), retired (73.3%), and married (96.0%). More than four-fifths (86.7%) of them were diagnosed with unstable angina, and the mean duration of the disease was 1.6 ± 2.0 months. The proportion of participants with hypertension, diabetes, and hyperlipidemia was 65.3, 37.3, and 28.0%, respectively. Most patients (61.3%) had at least two comorbidities. Additionally, 84% of patients reported an improved health status after PCI, and their mean age was 64.5 ± 8.5 years. Most were male (58.7%), retired (74.6%) and married (96.8%). Furthermore, 16% of patients reported no change in their health status, and their mean age was 62.2 ± 10.9 years, and they were mostly male (83.3%), retired (66.7%), and married (91.7%).

**Table 1:** Socio-demographic and clinical characteristics of CHD patients.

Characteristic	Overall (*n* = 75) *N* (%) or Mean ± SD	Improved (*n* = 63) *N* (%) or Mean ± SD	No change (*n* = 12) *N* (%) or Mean ± SD
Socio-demographic			
Gender			
Male	47 (62.7)	37 (58.7)	10 (83.3)
Female	28 (37.3)	26 (41.3)	2 (16.7)
Age (years)			
Mean ± SD	64.6 ± 9.1	64.5 ± 8.5	62.2 ± 10.9
Range	39–84	41–84	39–82
Educational level			
Illiteracy or primary school	8 (10.7)	8 (12.7)	0 (0)
Secondary school	33 (44.0)	26 (41.3)	7 (58.3)
High school or technical secondary school	22 (29.3)	18 (28.6)	4 (33.3)
University degree and above	12 (16.0)	11 (17.5)	1 (8.3)
Occupation			
Working^a^	20 (26.7)	16 (25.4)	4 (33.3)
Retired	55 (73.3)	47 (74.6)	8 (66.7)
Marital status			
Married	72 (96.0)	61 (96.8)	11 (91.7)
Unmarried^b^	3 (4.0)	2 (3.2)	1 (8.3)
Monthly income (Chinese Yuan, CNY)			
≤4,000	37 (49.3)	32 (50.8)	5 (41.7)
>4,000	38 (50.7)	31 (49.2)	7 (58.3)
Smoking			
Yes	13 (17.3)	11 (17.5)	2 (16.7)
No	62 (82.7)	52 (82.5)	10 (83.3)
Drinking			
Yes	13 (17.3)	12 (19.0)	1 (8.3)
No	62 (82.7)	51 (81.0)	11 (91.7)
Exercise			
Yes	26 (34.7)	22 (34.9)	4 (33.3)
No	49 (65.3)	41 (65.1)	8 (66.7)
Clinical characteristics			
CHD type			
Stable angina	1 (1.3)	0 (0)	1 (8.3)
Unstable angina	65 (86.7)	55 (87.3)	10 (83.3)
Acute myocardial infarction	9 (12.0)	8 (12.7)	1 (8.3)
Duration of CHD (months)			
Mean ± SD	1.6 ± 2.0	1.7 ± 2.1	0.8 ± 1.0
Range	0–12	0–12	0–1.5
Disease state			
First episode	41 (54.7)	32 (50.8)	9 (75.0)
Relapse	34 (45.3)	31 (49.2)	3 (25.0)
Comorbidities			
Yes	67 (89.3)	56 (88.9)	11 (91.7)
No	8 (10.7)	7 (11.1)	1 (8.3)
Number of comorbidities			
≤1	29 (38.7)	27 (42.9)	2 (16.7)
2	23 (30.7)	19 (30.2)	4 (33.3)
≥3	23 (30.7)	17 (27.0)	6 (50.0)
Prevalence of comorbidities			
Hypertension	49 (65.3)	42 (66.7)	7 (58.3)
Diabetes	28 (37.3)	21 (33.3)	7 (58.3)
Hyperlipidemia	21 (28.0)	18 (28.6)	3 (25.0)

^a^Working includes working in public institutions and companies, civil servants and freelancers. ^b^Unmarried includes single, divorced, widowed and separate.

Table 2 describes the score distribution of the EQ-5D-5L HSU and SAQ at both time points. The mean EQ-5D-5L HSU scores at baseline and follow-up were 0.850 and 0.975, respectively, with an average change of 0.125 (*p* < 0.001). For the SAQ, the mean scores were 56.414 and 72.077 at the two time points, respectively, with an average change score of 15.663 (*p* < 0.001).

**Table 2:** Summary statistics of EQ-5D-5L HSU and SAQ (*N* = 75).

Instrument scale	Baseline				Follow-up				Average change
	Mean ± SD	Median	LQ	UQ	Mean ± SD	Median	LQ	UQ	Mean ± SD
EQ-5D-5L HSU	0.850 ± 0.147	0.897	0.779	0.951	0.975 ± 0.102	1.000	1.000	1.000	0.125 ± 0.117^*^
SAQ	56.414 ± 10.149	56.322	48.280	64.368	72.077 ± 6.377	73.563	68.966	75.862	15.663 ± 9.387^*^

LQ, lower quartile; UQ, upper quartile; SD, standard deviation; EQ-5D-5L, five-level EuroQoL five-dimensional questionnaire; HSU, health state utility; SAQ, Seattle angina questionnaire; ^*^Denotes change at a statistical significance level of *p* < 0.001 at 0.05 confidence level.

### 3.2. Responsiveness

Table 3 reports the responsiveness results of the EQ-5D-5L. Among all patients, the EQ-5D HSU increased by 0.125 (*p* < 0.001) after PCI. The ES of EQ-5D-5L HSU was 0.850, indicating large responsiveness in all patients. Among patients who responded to the anchor transition as “improvement” (including minimal improvement and much improvement), the ES was 1.152, suggesting a large effect size. Additionally, for the patients who were “no change,” the change of HSU was 0.028 (*p* > 0.05). ES showed no responsiveness among the “no change” group.

**Table 3:** Responsive to SAQ of the EQ-5D-5L HSU at four-week after PCI.

Variables	EQ-5D-5L HSU (Mean ± SD)		
	All (*n* = 75)	No change (*n* = 12)	Improved (*n* = 63)
Baseline score	0.850 ± 0.147	0.898 ± 0.233	0.841 ± 0.125
Follow-up score	0.975 ± 0.102	0.927 ± 0.239	0.985 ± 0.040
Change score	0.125 ± 0.117^*^	0.028 ± 0.044	0.144 ± 0.118^*^
ES	0.850	0.120	1.152

SAQ, Seattle angina questionnaire; EQ-5D-5L, five-level EuroQoL five-dimensional questionnaire; HSU, health state utility; ES, effect size.^*^Denotes correlation at a statistical significance level of *p* < 0.01. The “No change” group and “Improved” group were divided by anchor (SAQ).

### 3.3. Minimal clinically important difference

#### 3.3.1. Anchor-based analysis

There was a moderate correlation between the change scores of the EQ-5D HSU and SAQ, with a correlation coefficient of 0.533 (Table 4). The MCID estimates of the EQ-5D HSU based on the SAQ-anchored method are listed in Table 5. Based on the SAQ scores, no participant reported a worsening health state from baseline to four-week after PCI; therefore, in this study, we only focused on the improved MCID. As shown in Table 5, an increase of 0.052 points (95% CI: −0.061, 0.165) in the EQ-5D HSU corresponded to a minimally important change for patients anchored by the SAQ.

**Table 4:** The Spearman correlation between EQ-5D-5L HSU and SAQ.

	EQ-5D-5L HSU (baseline)	EQ-5D-5L HSU (follow-up)	EQ-5D-5L HSU (change)	SAQ (baseline)	SAQ (follow-up)	SAQ (change)
EQ-5D-5L HSU (baseline)	1.00					
EQ-5D-5L HSU (follow-up)	0.306^**^	1.00				
EQ-5D-5L HSU (change)	−0.898^**^	0.001	1.00			
SAQ (baseline)	0.679^**^	0.241^*^	−0.566^**^	1.00		
SAQ (follow-up)	0.398^**^	0.344**	−0.258^*^	0.399^**^	1.00	
SAQ (change)	−0.470^**^	0.029	0.533^**^	−0.822^**^	0.092	1.00

EQ-5D-5L, five-level EuroQoL five-dimensional questionnaire; HSU, health state utility; SAQ, Seattle Angina Questionnaire;^*^Denotes correlation at a statistical significance level of *p* < 0.05. **Denotes correlation at a statistical significance level of *p* < 0.01.

**Table 5:** Anchor-based MCID estimates.

SAQ category	Mean change ± SD	MCID (95%CI)
Much improvement	0.156 ± 0.120	0.076 (0.015, 0.167)
Minimal improvement	0.080 ± 0.085	**0.052** (−0.061, 0.165)
No change	0.028 ± 0.044	

SAQ, Seattle angina questionnaire; MCID, minimal clinically important difference; the value of bold text is the minimal clinical important difference of EQ-5D-5L HSU calculated by anchor-based method.

**Figure 1:**
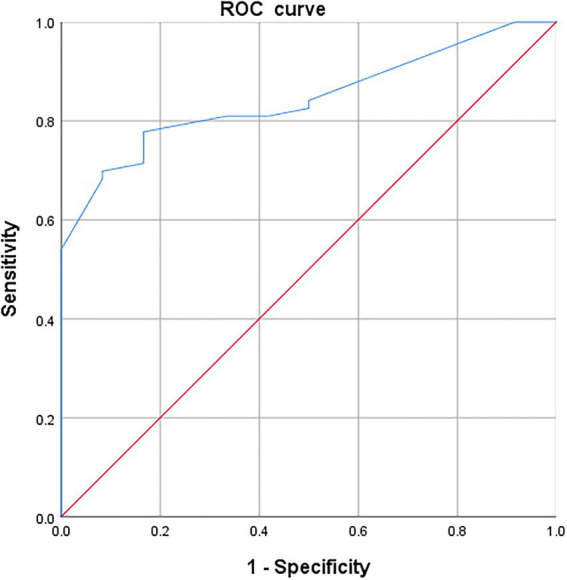
Receiver-operating characteristic (ROC) curve of EQ-5D-5L HSU change score in patients whose health states improved.

Receiver operating characteristic analysis was also performed to identify improved MCID only (Figure 1). The MCID estimate derived from the ROC curve was 0.098, corresponding to a sensitivity of 69.8% and a specificity of 91.7%. The AUC anchored by the SAQ was 0.845 (95% CI: 0.753, 0.936), suggesting that EQ-5D can excellently distinguish patients whose health states improved and those whose health states did not change.

#### 3.3.2. Instrument- and distribution-based analysis

Based on the instrument-defined method, the MCID of EQ-5D HSU was 0.055 (95% CI: 0.054, 0.58). An MCID estimate of 0.074 was produced using half the SD of HSU at baseline. The MCID derived from the SEM value was 0.078 (Table 6).

**Table 6:** Distribution-based and instrument-based MCID estimates.

EQ-5D-5L HSU	Distribution-based method				Instrument-based method
	SD	Half SD	One-SEM	Cronbach’s *α*	
Baseline	0.147	0.074	0.080	0.707	
Follow-up			0.076	0.731	
MCID		**0.074**	**0.078**		**0.055**

EQ-5D-5L, five-level EuroQoL five-dimensional questionnaire; HSU, health state utility; SD, standard deviation; SEM, standard error measurement; MCID, minimal clinically important difference; bold text denotes the MCID estimates calculated by each distribution-based method and instrument-based method.

### 3.4. Validity of the MCID estimates

Table 7 shows ratios of MCID estimates calculated by all methods to MDC_95%(ind)_ and MDC_95%(group)_. The ratios of MCIDs computed by all methods to MDC_95%(ind)_ were all less than 1, indicating that on individual levels, the MCIDs cannot be discriminated from measurement error to reflect a real meaningful change. However, the ratios of MCIDs to MDC_95%(group)_ all exceeded 1, which means that the MCID can detect the smallest important improvement in the group of 75 CHD patients with 95% confidence.

**Table 7:** Minimal clinically important difference (MCIDs) of the EQ-5D-5L HSU through different methods and the relation to the MDC at individual and group levels.

Variables	EQ-5D-5L HSU					
	MDC	half SD	One-SEM	Anchor-based method	ROC curve	Instrument-defined method
MCID		0.074	0.078	0.052	0.098	0.055
MDC_95%CI_						
Ind	0.216					
Group	0.025					
Ratio						
Ind		0.340	0.361	0.241	0.453	0.254
Group		2.948	3.124	2.083	3.926	2.203

EQ-5D-5L, five-level EuroQoL five-dimensional questionnaire; HSU, health state utility; MDC, minimal detectable change; SD, standard deviation; SEM, standard error measurement; ROC, receiver-operating characteristic; 95%CI, 95% confidence interval.

## 4. Discussion

This study estimated the responsiveness and MCID of the instrument in CHD patients after PCI surgery to support score interpretation and clinical application of the EQ-5D-5L. This study showed that the EQ-5D-5L was largely responsive to changes and provided evidence that the MCID of the EQ-5D-5L HSU in patients with CHD ranged from 0.052 to 0.098. The MCID calculated in this study can distinguish significant changes in the HSU at the group level but not at the individual level.

Responsiveness has been suggested as an additional criterion for evaluating HRQoL instruments, which can reflect the ability of an instrument to respond to changes and is essential for longitudinal validity (55). Various methods can be used to assess responsiveness, such as ES, SRM, relative efficiency (RE), and regression models. However, there is no consensus on the preferred method, and different methods provide different results (40, 41). According to Husted et al. (42) ES is one of the most frequently used approaches for assessing responsiveness. Therefore, this method was used in this study, and a large responsiveness of the EQ-5D-5L was found in all participants and improved participants.

The responsiveness of EQ-5D-5L HSU has been explored in several diseases. In patients with acute asthma who self-reported improvement, researchers found that the EQ-5D-5L had moderate to large responsiveness (56). Chen et al. (29) found that the EQ-5D-5L had small-to-moderate responsiveness in subacute and chronic stroke patients. Golicki et al. (57) found moderate to large responsiveness of the EQ-5D-5L in acute stroke patients whose health status improved from baseline. Although the above studies all found that EQ-5D-5L is responsive, this is quite different from our finding of large responsiveness. The source of inconsistency may be attributed to differences in patients’ disease stage or severity at baseline. In this study, 98.7% of patients had unstable angina or acute myocardial infarction, and 61.4% had at least two comorbidities, making patients have a stronger perception of the change in their health status compared with patients with chronic or stable disease stages, leading to large responsiveness. Furthermore, EQ-5D is more responsive to large treatment effects (28). Compared with other treatments, PCI surgery can greatly improve patients’ perceived HRQoL in the short term (58).

There is no “one-size-fits-all” method and no consensus on the best method to calculate the MCID of HRQoL instruments (59). Anchor-based and distribution-based methods are the main methods for evaluating the MCID (60). The anchor-based approaches use an external and independent indicator to calculate the MCID by comparing the scores in anchor-based groups, including the change difference method, ROC curve, regression model, etc. (44). In contrast, the distribution-based methods rely on measures of outcome variability, using the statistical property of the data set to identify the MCID (61), including the SD, SEM, and ES methods. Usually, these two methods are used together to ensure the accuracy of MCID estimates because each has its advantages and disadvantages (47). In this study, we chose the most commonly used anchor-based methods (i.e., the change difference method and ROC curve) and distribution-based methods (i.e., 0.5 SD and SEM) to calculate the MCID of the EQ-5D-5L (27, 62). In addition to these two methods, Luo et al. (50) first used an instrument-based method to evaluate MCID for the EQ-5D-3L, and the results showed that it is feasible to use this method to evaluate the MCID of preference-based HRQoL instruments. Subsequently, the instrument-based method has been widely used for calculating the MCID of the EQ-5D (46, 63, 64). Therefore, we used the above three methods simultaneously to ensure the accuracy of the MCID and found that the average MCID was 0.071, with a range from 0.052 to 0.098.

The ratios of MCIDs to MDC_95%(group)_ were all greater than 1, indicating that the MCIDs were valid at the group level and could be distinguished from measurement errors. However, the ratios of MCIDs to MDC_95%(ind)_ were all less than 1, which means that the calculated MCIDs would not be useful at the individual level. One possible explanation may be that most patients had longer CHD duration, and nearly half of them were not first-episode CHD, which made them adapt to this disease and improved baseline scores. Another explanation may be that most patients have at least two comorbidities, resulting in lower psychological expectations of health changes.

Previous studies commonly used the Global Rating of Change Questionnaire (GRCQ) as an anchor. However, the GRCQ contains only one question, which makes it difficult or impossible to capture changes in participants’ HRQoL (49). Moreover, its validity and reliability are uncertain (18). Studies have shown that disease-specific questions have higher construct validity than global transition questions as anchors for determining MCID (65). The GRCQ was not used or adopted in this study because of these limitations. In contrary, we used a valid, reliable, multi-attribute, and disease-specific instrument to anchor the EQ-5D-5L HSU as in previous studies (32, 66). Furthermore, it has been illustrated that responsiveness and MCID may be affected by the direction of changes (60, 67–69). However, no patients in this study reported that they experienced health deterioration after PCI anchored by SAQ change scores. The outcome of clinical interest lies in surgery-associated health improvement and the resulting effect on CHD patients’ quality of life rather than exacerbation. Therefore, deteriorative responsiveness and MCID were not evaluated in this study.

The MCID values may vary according to disease, interventions, and baseline characteristics, including socio-demographic and clinical characteristics; therefore, MCID may vary by gender, education level, type of CHD, and different comorbidities in this study (47, 70). In particular, studies found that the prognosis of male patients with CHD after PCI was better than that of female patients; the mortality, incidence of major adverse cardiovascular events, and bleeding events after PCI in female patients were higher than those in male patients; and the health status (such as HRQoL, mental health) of female patients after PCI was worse than that of male patients (71–73). Therefore, these differences in prognosis between genders may affect their perception of health and cause the MCID to vary among genders. However, due to the limited sample size within anchor-defined categories for the different stratified subgroups, it was impossible to explore whether the resultant MCID estimates based on these clinical/demographic factors in patients with CHD were different. We suggest exploring this issue using a larger sample size in the future.

This is the first study to evaluate the responsiveness and MCID of the EQ-5D-5L HSU in CHD patients after PCI. This study has several advantages. First, in addition to commonly used anchor-based and distribution-based methods, we adopted the instrument-based method to ensure the accuracy of the MCID estimates. Second, we used a multidimensional disease-specific scale as an anchor, which can reflect the changes in patients’ HRQoL from multiple dimensions. Third, we analyzed whether the MCID calculated by each method could reflect meaningful changes at both the individual and group levels, which allowed us to avoid false interpretations of the MCID.

This study also has few limitations. First, the patients were recruited from one Tianjin city hospital, which may not represent all the CHD patients across China. Second, the small sample size of our study may have affected the accuracy of the results, although it met the basic requirements for calculating the MCID (26). Third, the responsiveness and MCID may differ depending on the research setting, including interventions and patients’ characteristics. Therefore, the results of this study may not be applicable under other conditions. Fourth, this was a longitudinal real-world study, and randomized controlled trials are needed to verify the MCID among CHD patients after PCI in the future.

## 5. Conclusion

The EQ-5D-5L was largely responsive to patients with CHD undergoing PCI surgery. The MCID of EQ-5D-5L was 0.071, with a range between 0.052 and 0.098 in this study, and the calculated MCIDs could only determine whether patients experienced meaningful changes at the group level. Future studies should focus on the calculation of deteriorative responsiveness and MCID and examine the validity of MCID in CHD patients at individual levels.

## Data availability statement

The raw data supporting the conclusions of this article will be made available by the authors, without undue reservation.

## Ethics statement

The studies involving human participants were reviewed and approved by the Ethics Review Board of the School of Health Care Management, Shandong University (Reference No. ECSHCMSDU20191002). The patients/participants provided their written informed consent to participate in this study.

## Author contributions

LD and SL designed the study. QF performed the data collection. LD and YZ performed the data analyses, and all authors contributed to interpreting the data. YZ drafted the manuscript, which was critically revised by all others. All authors read and approved the final manuscript.

## Funding

Financial support for this project was provided by NHC Key Lab of Health Economics and Policy Research (Shandong University; no. NHC-HEPR2018003) and National Natural Science Foundation of China (no. 71403056).

## Conflict of interest

The authors declare that the research was conducted in the absence of any commercial or financial relationships that could be construed as a potential conflict of interest.

## Publisher’s note

All claims expressed in this article are solely those of the authors and do not necessarily represent those of their affiliated organizations, or those of the publisher, the editors and the reviewers. Any product that may be evaluated in this article, or claim that may be made by its manufacturer, is not guaranteed or endorsed by the publisher.
